# Different management of adrenocortical carcinoma in children compared to adults: is it time to share guidelines?

**DOI:** 10.1007/s12020-021-02874-z

**Published:** 2021-09-24

**Authors:** Salvatore Grisanti, Deborah Cosentini, Marta Laganà, Antonella Turla, Alfredo Berruti

**Affiliations:** Medical Oncology Unit, Department of Medical and Surgical Specialties, Radiological Sciences and Public Health, University of Brescia at ASST Spedali Civili, Brescia, Italy

**Keywords:** Adrenocortical carcinoma, Pediatric, Chemotherapy

## Abstract

Pediatric and adult adrenocortical carcinomas differ in many respects but treatment is often similar in both age groups. The *Journal of Clinical Oncology* recently published the results of a risk-stratified single-arm interventional trial conducted by the Children’s Oncology Group in which 77 patients were treated in three different interventional cohorts. In this Point of View paper we comment on the treatment strategies adopted within the ARAR0332 trial in terms of surgery approach, duration of adjuvant therapies, and palliative chemotherapy. We focus on the differences in the treatment of pediatric ACC patients compared to the ESE/ENSAT and ESMO guidelines released in 2018 for adult patients. For example, patients in stratum 3 and 4 received 8 (instead of 6) cycles of EDP chemotherapy but 8 months (instead of 24) of mitotane adjuvant therapy. Bearing clearly in the mind that pediatric and adult ACC patients represent different settings, we wonder whether there could be some areas of intervention overlapping to constitute a continuum of disease across ages. Thus, pediatric and adult cohoperative groups should be encouraged to collaborate in order to reach common guidelines for the treatment of such a rare disease.

Adrenocortical carcinoma (ACC) is an endocrine neoplasm arising in the outer part of the adrenal gland. Incidence of ACC has a bimodal distribution with two peaks in the first and fifth decades of life [1]. Adult ACC is a rare cancer with a reported incidence of 0.7–2 cases per million people/year worldwide [2]. Pediatric ACC is even rarer with a reported incidence of 0.2–0.3 cases per million people/year worldwide [3–6] except for the cluster associated with the TP53-R337H pathogenetic variant identified in Southern Brazil that is 15 times more frequent than non-Brazilian ACC cases [7]. ACCs arising during childhood have distinct features from those of adult life. First, pediatric ACC more often arise in the context of a cancer predisposing familial genetic syndrome such as the Li–Fraumeni syndrome and the Beckwith–Wiedemann syndrome with known hereditable genetic alterations at the germinal line [8]. Second, approximately 90% of ACC in children are hyperfunctioning with clinical signs of sex hormones, cortisol, or aldosterone hypersecretion or mixed endocrine syndromes [8]. Most young patients present with virilization (pubic hair, accelerated growth, and skeletal maturation, an enlarged penis or clitoris, hirsutism, and acne) due to excess androgen secretion alone or in combination with cortisol in more than 80% of patients [9, 10]. Third, despite the clinical course is variable, outcome may be more favorable in children than in adult patient, younger age is associated with better outcomes, and long-term survivors are more frequent in children than in adult patients with the same stage of disease, accordingly with the different biological and clinical pattern of presentation [5, 11].

Because of its rarity, pediatric ACC has been less studied than the adult counterpart and most of the knowledge derives from studies conducted in the South Brazilian population. This has probably represented an obstacle for conducting clinical trials and for defining precise guidelines for clinical management of ACC in children. Therefore, whether ACC in children and adults should be treated differently or not remains a matter of active discussion.

With the above premises we have read with great interest the article by Rodriguez-Galindo et al. published in the April 2021 issue of the *Journal of Clinical Oncology* [12]. The authors reported the results of clinical management of 77 eligible patients with stage I–IV ACC treated in three different interventional cohorts within the Children’s Oncology Group ARAR0332 trial.

According to clinical stage, patients were addressed to receive surgery alone (stage I), surgery and retroperitoneal lymph node dissection (RLND) (stage II), surgery plus RLND plus EDP chemotherapy for eight cycles and mitotane for 8 months (stages III–IV). The 5-year event-free survival (EFS) and overall survival by clinical stages were respectively: 86.2 and 95.2% (stage I), 53.3 and 78.8% (stage II), 81 and 94.7% (stage III), and 7.1 and 15.6% (stage IV). The ARAR0332 trial showed that surgery alone was less effective in stage II ACC as opposed to stage I, whereas surgery followed by chemotherapy plus mitotane was associated with a good outcome in stage III but not in stage IV patients. In the last setting this multidisciplinary approach appeared not cost-effective. In addition, the combination of mitotane and chemotherapy prescribed in ARAR0332 resulted in significant toxicity.

We congratulate all authors and the Children’s Oncology Group for performing a complex trial in a rare and difficult disease that represents a key point in future studies of pediatric ACC; however, we would like to remark some considerations about differences between pediatric and adult treatment of ACC.

First, patients falling in stratum 2 (stage II, large tumors, normal postoperative hormonal levels) were treated with surgery and synchronous or metachronous RLND. These patients had poorer survival outcomes in terms of both EFS and OS than expected. It is probable that this cohort comprised a fraction of patients with functioning tumors and a higher Ki67 that are both known poor prognostic factors in adult ACC [13, 14]. Adult ACC with these characteristics are considered high risk and would be offered adjuvant mitotane treatment for 2 years according to 2018 ESE/ENSAT and ESMO guidelines [15–17]. It would have been interesting if the authors had separately analyzed the patient outcome statified by either Ki67 or preoperative hormonal status.

Second, all patients falling in stratum 3 (stage III, inoperable large tumors or R1 tumors, abnormal postoperative hormonal levels) or in stratum 4 (stage IV, metestatic disease) received front-line extensive surgery followed by eight EDP cycles plus mitotane. Debulking surgery in patients with aggressive malignant disease has a debatable impact on patient prognosis and delays the initiation of a potentially effective systemic antineoplastic treatment. We do believe that surgical removal of even large tumors made metabolically less active by systemic treatment can be advantageous [18]. Along this line, our strategy in adult and young adult patients is to administer four to six cycles of EDP regimen plus mitotane in stage III/IV adult ACC patients, followed by surgical treatment in case of achievement of objective response or disease stabilization and evaluating a tumor debulking greater than 90% of the total tumor mass [19]. This protocol allowed us to detect a pathological complete response in 7% of patients, which was associated with an extremely good prognosis.

Third, according to adult international guidelines, mitotane therapy in locally advanced/metastatic ACC is continued till disease progression in case of advanced disease and for at least 2 years in adjuvant setting [15, 16, 20]. This is in contrast with the ARAR0332 trial in which mitotane was interrupted after 8 months. Mitotane treatment for 8 months could be an undertreatment considering that approximately 4 months are required for attaining a therapeutic range in the plasma and that approximately 10% and up to 40% of patients never reach the therapeutic range [21].

Fourth, toxicity is an important issue in this category of patients and we wonder whether eight instead of six cycles of EDP do add a substantial benefit or only increase toxicity. Eight EDP cycles are in fact quite toxic, both in terms of cumulative bone marrow toxicity and peripheral neuropathy that last for a long time and greatly hamper the quality of life. Not to mention the late risk of congestive heart failure, due to the doxorubicin exposure close to the maximum tolerated dose (320 mg/sqm). Given that the majority of pediatric ACC occurs at a median age of 3–4 years and that fortunately these patients will outperform better that adult patients, the issue of long-term toxicity could represent an element of concern.

In conclusion, do pediatric and adult ACC represent separate entities that need different management?

The answer is still far to answer. However, as the authors comment in their introduction, treatment of pediatric ACC has been borrowed from adult ACC and several areas of overlap exist between pediatric and adult ACC management. Thus, we do believe that interaction between pediatric and adult cohoperative groups could be beneficial for both in drawing and following common guidelines for the treatment of ACC as a continuum across ages.
